# Exhausted due to the pandemic: Validation of Coronavirus Stress Measure and COVID-19 Burnout Scale in a Polish sample

**DOI:** 10.1007/s12144-021-02543-4

**Published:** 2021-11-26

**Authors:** Marcin Moroń, Murat Yildirim, Łukasz Jach, Justyna Nowakowska, Karina Atlas

**Affiliations:** 1 Institute of Psychology, University of Silesia, 40-126, Grazynskiego Street 53, Katowice, Poland; 2 Department of Psychology, Faculty of Science and Letters, Ağrı İbrahim Çeçen University, Ağrı, Turkey; 3 Department Neuroscience, Psychology and Behaviour, University of Leicester, Leicester, UK

**Keywords:** COVID-19 burnout, Coronavirus stress, Mental health problems, Resilience

## Abstract

This study validated Polish versions of the Coronavirus Stress Measure (CSM) and the COVID-19 Burnout Scale (COVID-19-BS) to measure stress and burnout associated with COVID-19. Participants were 431 Polish young adults (72.6% female; Mean_age_ = 26.61 ± 12.63). Confirmatory factor analysis verified a one-factor solution for both the CSM and the COVID-19-BS. Both scales had high internal consistency reliability. Coronavirus stress and COVID-19 burnout were positively related to depression, anxiety, and stress and negatively related to resilience. The coronavirus stress and COVID-19 burnout were correlated with elevated levels of depression, anxiety, and stress over and beyond resilience, age, and gender. Findings suggest that the Polish versions of the CSM and the COVID-19-BS are valid scales to measure stress and burnout related to COVID-19. Findings also demonstrated that the coronavirus stress and COVID-19 burnout experienced during the later stages of the pandemic might be a permanent risk factor for mental health problems.

## Introduction

The COVID-19 pandemic caused severe psychological distress in the general population (Cooke et al., 2020; Necho et al., 2021; Salari et al., 2020) and was an extremely stressful condition, particularly for health care professionals (Pappa et al., 2020) and vulnerable groups such as patients with chronic illness (Rajkumar, 2020). On average, around 40% of the general population suffered from psychological distress due to the COVID-19 pandemic (Necho et al., 2021). Early longitudinal studies conducted between January and March 2020 indicated no clinically significant longitudinal changes in stress, anxiety, and depression levels in the four-week interval (Wang et al., 2020). Other studies conducted between March and June 2020 yielded similar results in periods of two and three months (Brailovskaia et al., 2021; Somma et al., 2021). Stress experienced due to the pandemic was pointed to as a mediator of longitudinal effects of COVID-19 lockdowns of well-being (Achterberg et al., 2021). These results indicate that the stress related to the pandemic should be considered chronic and severe.

A psychological syndrome emerging as a prolonged response to chronic stressors is burnout (Maslach & Leiter, 2016). Burnout is assumed to involve a misfit between internal dispositions (i.e., the characteristics of the individual) and external conditions (i.e., the characteristics of the environment; Bianchi et al., 2021; Lazarus & Folkman, 1984). However, the concept was initially identified in the workplace context (Maslach et al., 2001), burnout, could appear also in other situations, including chronic stressors (Mikolajczak et al., 2017). The core dimensions of burnout are overwhelming exhaustion, feelings of cynicism and detachment from the job, a sense of ineffectiveness, and lack of accomplishment (Bianchi et al., 2021; Maslach & Leiter, 2016).

In the context of the pandemic, the risk of burnout was examined in health care workers during the pandemic, with 40–70% of participants screened positive (Barello et al., 2020; Denning et al., 2021; Jalili et al., 2021; Talaee et al., 2020). Parental burnout was a significant problem among 20% of parents during the pandemic (Marchetti et al., 2020). Fewer studies on the COVID-19 specific measurements of stress and burnout were conducted in the general public (Yildirim & Solmaz, 2020).

Persistent uncertainty regarding the spread of the virus, prolonged preventive measures, and changes in daily routines led to psychological problems such as anxiety, mental confusion, social deprivation, and depression (Yildirim & Arslan, 2020). These mental health problems were frequently identified as correlates or causes of psychological burnout (Ahola & Hakanen, 2014; Koutsimani et al., 2019). Several studies demonstrated that chronic stress related to the pandemic was associated with decreased psychological resources, which helps to cope with setbacks, challenges, disappointments, and failures related to the pandemic, such as resilience, optimism, psychological flexibility, and social connectedness (Arslan et al., 2020; Yıldırım et al., 2021; Yildirim & Solmaz, 2020).

To facilitate research and practice, Arslan et al. (2020) validated the Coronavirus Stress Measure (CSM) adapted from the perceived stress scale (Cohen et al. 1983) to assess the pandemic-related stress. The validation study showed that CSM had a unidimensional structure, high internal reliability, and good convergent validity (Arslan et al., 2020). Similarly, Yildirim & Solmaz (2020) validated the COVID-19 Burnout Scale (COVID-19-BS) adapted from the Maslach-Pines’s (2005) Burnout Measure-Short Version (BMS). In their study, the COVID-19-BS had a unidimensional factor structure, high internal consistency, and evidence of convergent validity with indicators of mental health. Moreover, resilience had a protective role against the COVID-19 stress and burnout (Yildirim & Solmaz, 2020). The study conducted among health care staff by Yildirim et al., (2021) showed that the COVID-19 burnout was lower among highly optimistic individuals and those who experience better social connectedness. Moreover, the study showed that both optimism and social connectedness mediated the relationship between coronavirus stress and depressive symptoms.

The goal of the current study was to validate the Polish versions of the CSM (Arslan et al., 2020) and the COVID-19-BS (Yildirim & Solmaz, 2020). In order to test the validity of the CSM and the COVID-19-BS scales, we conducted confirmatory factor analyses and internal consistency analyses. We expected that the CSM and COVID-19-BS would yield a one-factor solution with high internal consistency reliability. Analogically to the original study (Yildirim & Solmaz, 2020), we also tested associations of CSM and the COVID-19-BS with resilience.

Moreover, we assessed relationships between the Coronavirus stress, the COVID-19 burnout, depression, anxiety, and stress. We expected that individuals experiencing a higher level of stress and burnout due to the pandemic would report higher depression, anxiety, and stress. Yildirim & Solmaz (2020) examined the COVID-19 burnout in the relatively early stages of the pandemic. The current study was conducted more than a year after the pandemic outbreak in Poland. Therefore, it examines burnout not only in terms of the immediate consequence of stress connected with disruptions of daily functioning (e.g., job, education, relationships) in the first months of the pandemic but also as a result of long-lasting chronic stressors caused by the pandemic (e.g., prolonged social isolation, worsened mental health). The restrictions put in place to contain the COVID-19 virus (e.g. lockdown and long deprivation of school) may be particularly difficult for adolescents, who rely heavily on their peer connections for emotional support (Magson et al., 2020). Thus, we also examined the differences in coronavirus stress and burnout between adolescents and adults.

## Method

### Participants

A convenience sample of 431 participants (72.6% female; age ranged from 16 to 82 with a mean age of 26.61 years, SD = 12.63) was drawn from the general public of Poles using an online survey. The age range of adolescence was determined according to recent reviews (Newby et al., 2021), namely as age between 10 and 19 years. One hundred and six participants were adolescents and three hundred twenty-five were adults. They mainly belonged to average socioeconomic status (76.6%). Of the participants, 78.2% had no history of chronic disease. Seventy-five participants were married (17.4%), 142 were in an informal romantic relationship (32.9%), 185 were singles (42.9%), five were divorced (1.2%), and 24 individuals did not report their status or described their relationship as other (e.g., betrothal; 5.6%). Among all respondents, 61 participants reported confirmed history of COVID-19 (14.2%), and 139 suspected that they were infected but not verified by a test (33.3%). Among the participants, 72.6% reported that at least one person from their family members had been confirmed with COVID-19. The sample size was appropriate to conduct confirmatory factor analysis and to ensure power to detect small effect sizes in structural equation models (Wolf et al., 2013).

## Measures

### COVID-19 Burnout Scale (COVID-19-BS)

The COVID-19-BS consists of 10 items adapted from the Burnout Measure-Short Version (Malach-Pines, 2005). Yildirim & Solmaz (2020) modified the items by replacing references to “your work” in the wording of the original items with “COVID-19” (see Table 2). The items were translated into Polish by two psychologists fluent in English and then back-translated by two professional proofreaders with experience in psychological literature. The back-translated version was approved by the Author of the original study. Each item is rated on a 5-point Likert scale of 1 (*never*) to 5 (*always*). A total score is calculated by summing all 10 items, and a higher score indicates a higher level of burnout related to COVID-19.

### Coronavirus Stress Measure (CSM)

The CSM (Arslan et al., 2020) measures COVID-19 related stress and includes five items (see Table 1). Each item is rated on a 5-point Likert scale from 0 (*never*) to 4 (*very often*). Higher total scores indicate a higher level of stress related to COVID19. In the current study, we used a Polish version of the measure approved by the Author of the original study following the procedure described for the COVID-19-BS.

**Table 1:** Descriptive statistics, reliability, and factor loadings of the CSM items

Item	M	SD	I-T	CID	CFA loading
1. How often have you been upset because of the COVID19 pandemic? / Jak często zdarzało ci się być zdenerwowanym z powodu pandemii COVID-19?	2.615	1.106	.678	.846	.802
2. How often have you felt that you were unable to control the important things in your life due to the COVID19 pandemic? / Jak często zdarzało ci się czuć, że nie byłeś zdolny kontrolować ważnych rzeczy w swoim życiu z powodu pandemii COVID-19?	2.450	1.209	.652	.852	.702
3. How often have you felt nervous and stressed due to the COVID19 pandemic? / Jak często zdarzało ci się czuć się nerwowym i zestresowanym z powodu pandemii COVID-19?	2.550	1.164	.728	.834	.854
4. How often have you found that you could not cope with all the things that you had to do due to the COVID19 pandemic? / Jak często, z powodu pandemii COVID-19, zdarzało ci się zauważyć, że nie mogłeś poradzić sobie ze wszystkimi sprawami, które masz do zrobienia?	2.295	1.281	.713	.837	.668
5. How often have you felt difficulties were piling up so high that you could not overcome them due to the COVID19 pandemic? / Jak często, z powodu pandemii COVID-19, zdarzało ci się czuć, że trudności piętrzyły się tak bardzo, że nie mogłeś ich przezwyciężyć?	2.060	1.278	.704	.839	.653

### Brief Resilience Scale (BRS)

The BRS (Smith et al., 2008; for the Polish version see: Konaszewski et al., 2020) consists of 6 items assessing the ability to bounce back or recover from stress, to adapt to stressful circumstances, to not become ill despite significant adversity, and to function above the norm despite stress or adversity (e.g., “I tend to bounce back quickly after hard times.”). Each item is rated on a 5-point scale from 1 (*strongly disagree*) to 5 (*strongly agree*). The overall BRS score is estimated by reverse scoring three items and then adding the scores of all six items. Higher scores on the BRS indicating greater resilience. The reliability of BRS was 0.85 in the current study.

### Depression, Anxiety, and Stress Scale (DASS-21)

The DASS-21 (Antony et al., 1998; Lovibond & Lovibond, 1995) consists of 21 items assessing the symptoms of depression, anxiety, and stress. Each item is rated on a 4-point Likert scale from 0 (*Does not apply to me at all*) to 3 (*Applies to me very much or most of the time*). Higher scores indicating more negative experiences in the past week. This study used a Polish translation of DASS-21 (Makara-Studzińska, et al (n.d.), which showed good psychometric properties in previous studies in the Polish context (Scholten et al., 2017).

In the current study, we additionally used the cut-off criteria of severe depression (scores ≥ 21), anxiety (scores ≥ 15), and stress (scores ≥ 26) in order to examine an incremental validity of the COVID-19 burnout in predicting the clinically relevant intensity of psychological symptoms (Lovibond & Lovibond, 1995; see Juchnowicz et al., 2021).

## Procedure

The study was conducted online among adult Polish participants. Participation in the study was anonymous and voluntary. No monetary compensation was provided. The participants were recruited using a convenience sampling technique and an online survey. The participants were invited to participate in the study by invitations posted on social media and by recruiters who were undergraduate students in late April and May 2021. Before filling the questionnaire, the participants were informed about the terms of participation (e.g., the opportunity to stop the study whenever they decided to do so) and gave their informed consent. This study was approved by the Institutional Review Board (decision number: KEUS 115/04.2021).

## Data analysis

The factor structures of the CSM and COVID-19-BS were verified by confirmatory factor analysis (CFA) using maximum likelihood (ML) estimation. The cut-off criteria for goodness of fit indices in SEM were the comparative fit index (CFI) > 0.95, the Tucker-Lewis index (TLI) > 0.95, the root mean square error of approximation (RMSEA) < 0.06, and root mean square residual (SRMR) < 0.08 (Hu & Bentler, 1999). Correlations between the measured variables were explored using Pearson product-moment test. We also used logistic regression to test whether the CSM and COVID-19-BS have incremental validity in predicting mental health problems over and beyond gender, age, and resilience.

## Results

### The Factor Structure of the Polish Version of CSM

Descriptive statistics form the items of CSM are given in Table 1.

The initial CFA indicated that one-factor solution provided a poor fit to the data (χ = 113.996; *df* = 5; *p* < 0.001; CFI = 0.898; TLI = 0.795; RMSEA = 0.225; 90% CI = [0.190 — 0.262]; SRMR = 0.050). Inspection of modification indices pointed out the covariance between items 4 and 5. Following this procedure, the model significantly improved by indicating a good fit to the data (χ = 14.086; *df* = 4; *p* = 0.007; CFI = 0.991; TLI = 0.976; RMSEA = 0.076; 90% CI = [0.036—0.122]; SRMR = 0.023). The CFA loading were between 0.653 (item 5) and 0.854 (item 3).

#### The Factor Structure of the Polish Version of the COVID-19-BS

Descriptive statistics for the items of COVID-19-BS are given in Table 2.

**Table 2:** Descriptive statistics, reliability, and factor loadings of the COVID-19-BS items

Item	M	SD	I-T	CID	CFA loadings
1. When you think about COVID-19 overall, how often do you feel tired? / Jak często czujesz się zmęczony, gdy myślisz ogólnie o COVID-19?	3.585	1.332	.661	.870	.701
2. When you think about COVID-19 overall, how often do you feel disappointed with people? / Jak często czujesz się rozczarowany ludźmi, gdy myślisz ogólnie o COVID-19?	3.638	1.155	.405	.887	.431
3. When you think about COVID-19 overall, how often do you feel hopeless? / Jak często masz poczucie beznadziejności, gdy myślisz ogólnie o COVID-19?	3.332	1.265	.750	.864	.818
4. When you think about COVID-19 overall, how often do you feel trapped? / Jak często masz poczucie uwięzienia, gdy myślisz ogólnie o COVID-19?	3.515	1.268	.665	.870	.764
5. When you think about COVID-19 overall, how often do you feel helpless? / Jak często czujesz się bezradny, gdy myślisz ogólnie o COVID-19?	3.471	1.297	.734	.865	.826
6. When you think about COVID-19 overall, how often do you feel depressed? / Jak często czujesz się przygnębiony, gdy myślisz ogólnie o COVID-19?	3.267	1.280	.768	.862	.852
7. When you think about COVID-19 overall, how often do you feel physically weak/sickly? / Jak często czujesz się fizycznie słaby/chorowity, gdy myślisz ogólnie o COVID-19?	2.439	1.254	.656	.871	.607
8. When you think about COVID-19 overall, how often do you feel worthless/like a failure? / Jak często czujesz się bezwartościowy/jak ofiara losu, gdy myślisz ogólnie o COVID-19?	2.401	1.360	.654	.871	.641
9. When you think about COVID-19 overall, how often do you feel difficulties sleeping? / Jak często miewasz problemy ze snem, gdy myślisz o COVID-19?	1.865	1.220	.531	.879	.454
10. When you think about COVID-19 overall, how often do you feel “I’ve had it”? / Kiedy myślisz o COVID-19 ogólnie, jak często czujesz "Miałem go"?	2.552	1.349	.359	.893	.329

I-T: Corrected item-total correlation; CiD: Cronbach’s alpha if item deleted; CFA loadings represent standardized estimates

The CFA indicated that one-factor solution provided a poor fit to the data (χ = 305.395; *df* = 35; *p* < 0.001; CFI = 0.871; TLI = 0.835; RMSEA = 0.134; 90% CI = [0.120 — 0.148]; SRMR = 0.065). Based on the modification indices, we drew covariance between item 7–8, item 8–9, item 7–9, and item 2–3. Following this procedure, the model significantly improved by indicating a good fit to the data (χ = 116.521; *df* = 31; *p* < 0.001; CFI = 0.959; TLI = 0.941; RMSEA = 0.080; 90% CI = [0.065 0.096]; SRMR = 0.041). CFA loadings ranged from 0.329 (item 10) to 0.852 (item 6).

#### Associations Between the Coronavirus Stress, COVID-19 Burnout, and Psychological Symptoms

Findings from the correlation analysis (see Table 3) indicated that CSM and COVID-19-BS were positively related to depression, anxiety, and stress and negatively related to resilience.

**Table 3:** Means, standard deviations, reliability and intercorrelations of the study variables

Variable	1	2	3	4	5	6
1. COVID-19 burnout						
2. COVID-19 stress	.785***					
3. Resiliency	-.377***	-.421***				
4. Depression	.582***	.610***	-.539***			
5. Anxiety	.578***	.573***	-.450***	.762***		
6. Stress	.693***	.729***	-.482***	.780***	.758***	
*M*	30.065	11.970	17.258	17.026	12.506	24.107
*SD*	8.964	4.901	5.321	12.314	11.658	11.578
α	.89	.87	.85	.91	.89	.90

^*^ *p* < .05; ** *p* < .01; *** *p* < .001

The Coronavirus stress and COVID-19 burnout did not correlated significantly with age (*r* = -0.039; *p* = 0.422; *r* = 0.033; *p* = 0.500, respectively). The analysis indicated that adolescents did not differ from adults in coronavirus stress (*M* = 11.717; *SD* = 4.647 vs. *M* = 12.052; *SD* = 4.985) and COVID-19 burnout (*M* = 29.066; *SD* = 8.648 vs. *M* = 30.391; *SD* = 9.053; *t* < 1.322; *p* > 0.187; Cohen’s *d* < 0.148). Regarding other indicators of mental health, the obtained results showed that adolescents did not differ from adults in stress (*M* = 27.717; *SD* = 12.505 vs. *M* = 27.028; *SD* = 13.426), anxiety (*M* = 13.962; *SD* = 10.522 vs. *M* = 12.031; *SD* = 11.987) and resilience (*M* = 16.811; *SD* = 5.160 vs. *M* = 17.403; *SD* = 5.373; *t* < -1.483; *p* > 0.139; Cohen’s *d* < -0.166). However, adolescents reported higher levels of depression (*M* = 19.792; *SD* = 11.901) than adults (*M* = 16.123; *SD* = 12.329; *t* = -2.683; *p* = 0.008; Cohen’s *d* = -0.300).

The Coronavirus stress was related to gender (*t*(429) = -5.305; *p* < 0.001; Cohen’s *d* = -0.573) with higher scores reported by women (*M* = 12.716; *SD* = 4.685) and lower reported by men (*M* = 9.992; *SD* = 4.931). Also the COVID-19 burnout was related to gender (*t*(429) = -6.548; *p* < 0.001; Cohen’s *d* = -0.707) with higher scores reported by women (*M* = 31.772; *SD* = 8.334) and lower reported by men (*M* = 25.669; *SD* = 9.123). Being diagnosed with COVID-19 or suspecting being infected was important for the level of the Coronavirus stress (*F*(3, 427) = 98.259; p = 0.006; η2 = 0.029). Individuals who suspected that they were infected experienced higher stress (*M* = 12.827; *SD* = 4.509) than individuals who were not infected (M = 11.150; SD = 7.746; Tukey’s test p = 0.009), but showed similar intensity of burnout compared to individuals with confirmed infection (*M* = 12.197; *SD* = 5.767) and those who did not know (*M* = 13.400; *SD* = 4.453). Being diagnosed with COVID-19 or suspecting being infected was also important for the level of the COVID-19 burnout (*F*(3, 427) = 603.552; *p* < 0.001; η2 = 0.052). Individuals who suspected that they were infected experienced higher burnout (*M* = 32.360; *SD* = 7.647) than individuals who were not infected (*M* = 27.961; *SD* = 8.800; Tukey’s test *p* < 0.001), but showed similar intensity of burnout compared to individuals with confirmed infection (*M* = 31.131; *SD* = 11.147) and those who did not know (*M* = 32.040; *SD* = 7.469).

Three logistic regression models examined the predictive validity of coronavirus stress and the COVID-19 burnout in predicting severe levels of depression, anxiety, and stress according to cut-off points proposed by Lovibond and Lovibond (1995). Null models included gender, age, and resiliency. We measured whether entering the coronavirus stress and the COVID-19 burnout into the model will result in a significant change in variance explained by the regression model and whether the coronavirus stress and the COVID-19 will be significant predictors of the severe intensity of the examined psychological symptoms (see Table 4).

**Table 4:** Results of logistic regression for severe level of depression, anxiety, and stress

Predictor	Depression			Anxiety			Stress		
	*b*(SE)	OR	Wald	*b*(SE)	OR	Wald	*b*(SE)	OR	Wald
Age	-.020 (.011)	.980	3.357	-.012 (.011)	.988	1.190	-.029 (.011)	.972	7.001**
Gender	.150 (.154)	1.161	.951	.112 (.157)	1.118	.507	.212 (.147)	1.123	1.143
Resilience	-.137 (.028)	.872	24.558***	-.153 (.028)	.858	29.280***	-.145 (.027)	.865	5.361***
CSM	.163 (.044)	1.117	13.617***	.090 (.044)	1.094	4.165*	.133 (.042)	1.142	10.283***
COVID-19-BS	.084 (.025)	1.087	10.919***	.109 (.026)	1.115	17.279***	.098 (.025)	1.103	15.530***

CSM – Coronavirus stress measure; COVID-19-BS – the COVID-19 burnout scale; OR – odds ratio; SE – standard error. * *p* < .05; ** *p* < .01; *** *p* < .001

When entered into model, the coronavirus stress and the COVID-19 burnout explained additional variance over and beyond age, gender, and resilience in predicting severe intensity of depression (χ2 for change = 88.857; *p* < 0.001; Δ Negelkerke *R*^2^ = 0.276), of anxiety (χ2 for change = 74.435; *p* < 0.001; Δ Negelkerke *R*^2^ = 0.241), and of stress (χ2 for change = 140.454; *p* < 0.001; Δ Negelkerke *R*^2^ = 0.411). These results indicated that the coronavirus stress and the COVID-19 burnout have incremental validity over and beyond the lack of resiliency, age, and gender and might be independent risk factors for developing severe mental health conditions due to the pandemic.

**Fig. 1:**
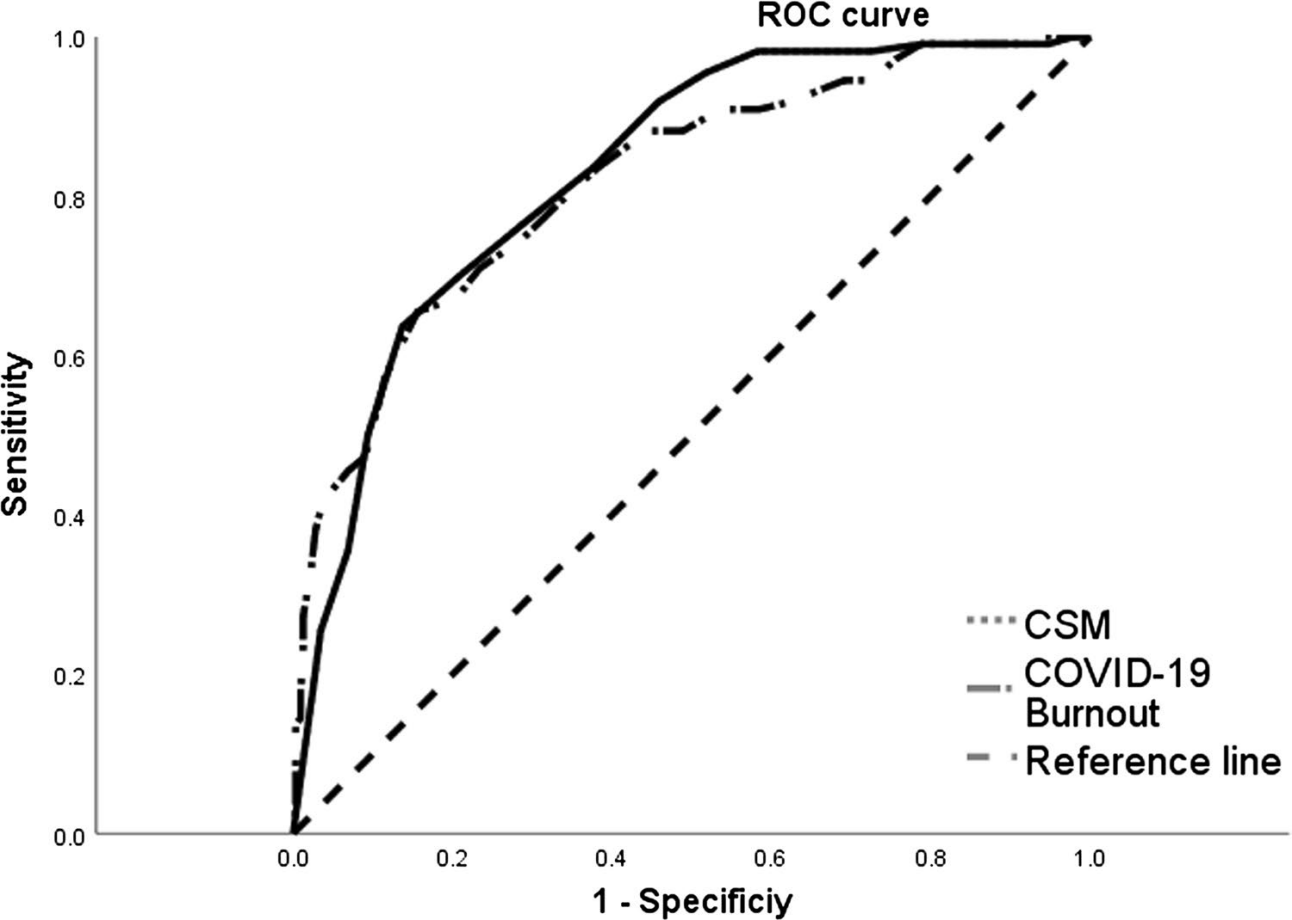
Receiver operating characteristic (ROC) curves for CSM and COVID-19-BS

Additionally, we conducted a receiver operating characteristic (ROC) analysis to determine cut-off points for coronavirus stress and the COVID-19 burnout, which might suggest the simultaneous presence of severe risk of depression, anxiety, and psychological distress. Among the participants, 110 were classified as at severe risk of these conditions (25.5%). The area under the curve was 0.83 for coronavirus stress, and 0.82 for the COVID-19 burnout was appropriate for the ROC analysis (Mandrekar, 2010). The ROC curves are given in Fig. 1.

Table 5 presents the sensitivity and specificity of coronavirus stress and COVID-19 burnout for potential cut-off points.

**Table 5:** Sensitivity and specificity of the detected cut points for simultaneous severe risk of depression, anxiety, and stress

Cut point	Coronavirus stress		Cut point	COVID-19 burnout	
	Sensitivity	Specificity		Sensitivity	Specificity
12.50	.836	.623	30.50	.836	.617
13.50	.764	.713	31.50	.800	.657
14.50	.700	.791	32.50	.745	.713
15.50	.636	.873	33.50	.709	.766

Since both measures validated in the present study might help screen for severe psychological consequences of maladaptive reactions to the pandemic, we decided to select cut-off points with higher sensitivity than specificity. Thus, we suggest 13.5 for coronavirus stress and 32.5 for COVID-19 burnout as temporary cut-off points, which might be helpful in the screening of risk of depression, anxiety, and stress due to the pandemic.

## Discussion

The COVID-19 pandemic established an unprecedented situation of chronic and highly stressful disruptions in daily routines, economy, and public health (Rajkumar, 2020; Necho et al., 2021). High intensity and chronicity of the COVID-19-related stress may lead to a syndrome of exhaustion, cynicism, and a sense of ineffectiveness and lack of accomplishment, which are characteristic symptoms of psychological burnout (Maslach & Leiter, 2016). The prevalence of burnout during the COVID-19 pandemic was examined among health personnel (Chen et al., 2020; Talaee et al., 2020), parents (Marchetti et al., 2020), and teachers (Pressley, 2021). However, less is known about COVID-19 burnout symptomatology in the general population. Therefore, the goal of the current study was to validate the Polish versions of the CSM and COVID-19-BS, which are convenient and easy to administer scales developed to assess the stress and burnout symptomatology among adults (Arslan et al., 2020; Yildirim & Solmaz, 2020).

The Polish version of the CSM (Arslan et al., 2020) demonstrated high internal consistency, unidimensional structure, and good convergent validity. The CFA loadings were significant and the highest for items referring to being nervous and stressed. The final structural model included covariation between items 4 and 5, which regarded difficulties in coping with stressors. The original and Polish versions of the CSM scale had a similar internal consistency and an identical, one-dimensional structure. These results indicate that the dynamics of experiences related to COVID-19 stress may be very similar, regardless of the cultural context. When comparing the CSM with other tools for studying stress related to the COVID-19 pandemic (Campo-Arias et al., 2020; Taylor et al., 2020), it should be noted that CSM is a relatively short measure, which may facilitate its use in batteries focused on studying more variables. From a different perspective, some other COVID-19 measures have a multi-dimensional structure that can measure the level of stress associated with specific aspects of a pandemic, while the CSM provides data on the overall stress level (Taylor et al., 2020).

The Polish version of the COVID-19-BS demonstrated unidimensional structure and high internal consistency. The CFA loadings indicated that feelings of depression, hopelessness, and helplessness were the most characteristic of the COVID-19 burnout. This finding is in line with the original study (Yildirim & Solmaz, 2020) and the proposition that exhaustion is the core aspect of burnout (Maslach & Leiter, 2016). Covariations added to the structural model based on the modification indices are in line with the findings of Malach-Pines (2005), pointing to a common source of variance for items regarding physical weakness (item 7) and sleep problems (item 9). Previous study results suggest that self-reported insomnia is a common problem in the general population of Poland (Nowicki et al., 2016), with the prevalence of sleep complaints about 50.5%. The low mean for item 9 might indicate that the participants did not identify the pandemic-related stress as the main factor affecting their sleep quality during the pandemic. Sleep problems are associated with lower self-esteem (Lemola et al., 2013), supporting covariance between items 8 and 9. Feeling hopeless was related to somatic symptoms (Ejdemyr et al., 2021), supporting the covariance between items 3 and 7.

The COVID-19 burnout correlated significantly with the COVID-19 stress, and the magnitude of this relationship was large. This result is in line with the findings that chronic stress related to or caused by the pandemic may result in burnout (see Maslach & Leiter, 2016). Since Yildirim and Solmaz (2020) measured the COVID-19 burnout a few months after the pandemic outbreak in 2020, the current results showed that after a year of the pandemic, the relationship between stress and burnout remains strong. Thus, individuals experiencing high stress due to the pandemic may constantly be in danger of developing burnout symptomatology.

The correlational analysis indicated that the coronavirus stress and the COVID-19 burnout were significantly related to depression, anxiety, and psychological distress. The obtained results were in line with studies on associations between burnout and depression (Bianchi et al., 2021) and between burnout and anxiety (Koutsimani et al., 2019). The causing factors of burnout, namely unresolvable and chronic stressors, generates a decrease in positive affective states and an increase in negative affective states, which have been identified as a basic depressogenic factor in individuals without other susceptibility factors to depression (Willner et al., 2013). Emotional exhaustion, depersonalization, and perceiving the situational demands as excessive may lead to higher anxiety (Ding et al., 2014). Chronic coronavirus stress might result in deterioration of the psychological resources such as psychological flexibility, social connectedness, and optimism (Yildirim et al., 2021), which might correspond to a higher propensity to experience emotional burnout due to the pandemic.

The present findings showed that the coronavirus stress and the COVID-19 burnout might be predictive for the severe intensity of depressive, anxiety, and stress symptoms. Among the participants, COVID-19 stress and burnout symptoms predicted severe levels of depression, anxiety, and stress over and beyond demographics and resiliency. The additional ROC curve analysis indicated initial cut-off points in COVID-19 stress (13.5) and burnout (32.5) measures which could be predictive in screening the risk of severe depression, anxiety, and stress symptoms in the general population. The findings of the current study indicated that the COVID-19 stress and burnout symptomatology might mainly result in the constant diminishing of capacity to deal with stress in daily life and its outcomes. Resiliency, considered a psychological ability to bounce back when struggling with stress (Smith et al., 2008), was negatively correlated with the COVID-19 stress and burnout, which demonstrated divergent validity of the COVID-19-BS.

The COVID-19 pandemic caused the occurrence of a large number of chronic stressors such as persistent fear of contagion, fear of economic or work difficulties, (mis)information overload, uncertainty and social isolation (Kira et al., 2021). The increased levels of stress associated with the pandemic can cause depression and anxiety due to impaired psychological control and deprived hope (Gallagher et al., 2021; Sher, 2020). In example, individuals experiencing occupational instability during the pandemic reported higher distress than those who were unemployed before the pandemic (Mimoun et al., 2020). Persistent stress can result in the neuropsychological changes, e.g. a chronic exposure to high cortisol levels which can in turn stimulate mesolimbic reward pathways within the brain resulting in maladaptive coping and further health consequences (Cianfarrini & Pampanini, 2021). Future studies should investigate longitudinally the impact of chronic stress associated with the COVID-19 which may lead to severe mental health outcomes particularly among groups at risk (e.g. among young adults; O’Connor et a., 2021).

The study showed that being diagnosed with COVID-19 or suspecting being infected was correlated with higher coronavirus stress and burnout. The results were consistent with previous findings about higher distress among people who had contact with individuals with a confirmed COVID-19 infection and those who had contact with an individual with suspected infection (Traunmüller et al., 2020). In both studies, suspected infection had psychologically the similar effect to actually being infected with COVID-19. Being infected may cause psychological distress due to the severity of the illness, actual health threat experienced due to the infection or postinfection physical discomforts (Cai et al., 2020). Suspecting infection may result in a hopelessness, loss of psychological control and persistent worries about health which can lead to elevated stress and burnout (Gallagher et al., 2021). Thus, the present study indicated that verification of patient’s infection status may help to prevent chronic coronavirus stress and provide appropriate psychological aid.

Findings from the current study support using the Polish version of the COVID19-BS as a brief, easy to administer, internally consistent, and unidimensional scale for assessing burnout related to the COVID-19 pandemic. The validity of the Polish version of the COVID-19-BS was confirmed by examining its associations with depression, anxiety, psychological distress, and resiliency. Due to the broad age range of the participants, the COVID-19-BS could be administered in the general population, but the generalizability of obtained findings is limited due to the non-representative sampling method used in the current study. Future studies may use the CSM and COVID-19-BS as a screening method in more extensive and more representative groups to establish cut-off criteria for severe burnout. Thanks to its brevity, the CSM and COVID-19-BS may be helpful in screening members of general population for potential risk of developing depression and anxiety symptomatology. However, prospective and longitudinal studies are needed to examine the long-term consequences of feelings of CSM and COVID-19 burnout. The future studies should also examine a test–retest reliability of the coronavirus stress and the COVID-19 burnout scales in order to test their temporal stability.

## Data Availability

The data and materials are available upon request from the corresponding author.

## Code Availability

The study code is available upon request from the corresponding author.
